# Extracts of *Brocchia cinerea* (Delile) Vis Exhibit In Vivo Wound Healing, Anti-Inflammatory and Analgesic Activities, and Other In Vitro Therapeutic Effects

**DOI:** 10.3390/life13030776

**Published:** 2023-03-13

**Authors:** Abdelkrim Agour, Ibrahim Mssillou, Azeddin El Barnossi, Mohamed Chebaibi, Amina Bari, Manal Abudawood, Yazeed A. Al-Sheikh, Mohammed Bourhia, John P. Giesy, Mourad A. M. Aboul-Soud, Badiaa Lyoussi, Elhoussine Derwich

**Affiliations:** 1 Laboratory of Natural Substances, Pharmacology, Environment, Modeling, Health, and Quality of Life, Faculty of Sciences Dhar El Mahraz, University Sidi Mohamed Ben Abdellah, Fez 30050, Morocco; 2 Laboratory of Biotechnology, Environment, Agrifood, and Health, Faculty of Sciences Dhar El Mahraz, University of Sidi Mohamed Ben Abdellah, Fez 30050, Morocco; 3 Biomedical and Translational Research Laboratory, Faculty of Medicine and Pharmacy of the Fez, University of Sidi Mohamed Ben Abdellah, BP 1893, Km 22, Road Sidi Harazem, Fez 30070, Morocco; 4 Department of Clinical Laboratory Sciences, College of Applied Medical Sciences, King Saud University, P.O. Box 10219, Riyadh 11433, Saudi Arabia; 5 Laboratory of Chemistry and Biochemistry, Faculty of Medicine and Pharmacy, Ibn Zohr University, Laayoune 70000, Morocco; 6 Toxicology Centre, University of Saskatchewan, Saskatoon, SK S7N 5B3, Canada; 7 Department of Veterinary Biomedical Sciences, University of Saskatchewan, Saskatoon, SK S7N 5B4, Canada; 8 Department of Integrative Biology, Michigan State University, East Lansing, MI 48824, USA; 9 Department of Environmental Sciences, Baylor University, Waco, TX 76706, USA

**Keywords:** analgesic activity, antibacterial, antioxidant, traditional medicine, wound healing

## Abstract

The plant *Brocchia cinerea* (Delile) (*B. cinerea*) has many uses in traditional pharmacology. Aqueous (BCAE) and ethanolic extracts (BCEE) obtained from the aerial parts can be used as an alternative to some synthetic drugs. In vitro, DPPH, FRAP and TAC are three tests used to measure antioxidant efficacy. Antibacterial activities were determined against one Gram positive and two Gram negative strains of bacteria. The analgesic power was evaluated in vivo using the abdominal contortion model in mice, while carrageenan-induced edema in rats was the model chosen for the anti-inflammatory test; wound healing was evaluated in an experimental second degree burn model. The results of the phytochemical analysis showed that BCEE had the greatest content of polyphenols (21.06 mg AGE/g extract), flavonoids (10.43 mg QE/g extract) and tannins (24.05 mg TAE/g extract). HPLC-DAD reveals the high content of gallic acid, quercetin and caffeic acid in extracts. BCEE has a strong antiradical potency against DPPH (IC_50_ = 0.14 mg/mL) and a medium iron reducing activity (EC_50_ = 0.24 mg/mL), while BCAE inhibited the growth of the antibiotic resistant bacterium, *P. aeruginosa* (MIC = 10 mg/mL). BCAE also exhibited significant pharmacological effects and analgesic efficacy (55.81% inhibition 55.64% for the standard used) and the re-epithelialization of wounds, with 96.91% against 98.60% for the standard. These results confirm the validity of the traditional applications of this plant and its potential as a model to develop analogous drugs.

## 1. Introduction

Plants have several pharmacological properties which are linked to the presence of various bioactive compounds, including among others, terpenoids, phenolic acids and flavonoids [1]. Several species of plants have been proposed as phytomedical to treat several diseases [2,3]. The family Asteraceae, or Composite, with the common names asters, daisies or sunflowers, contains a number of genera frequently used for therapeutic purposes. Species in this family contain a wide range of phenolic phytochemicals, essential oils, steroids and terpenoids [4].

Since ancient times, plants have been a source of substances used in the treatment of skin wounds. These can be burns caused by heat, electricity, cold, radiation and chemicals [5]. Wound healing requires a complex, orderly and dynamic process that proceeds through the following stages: coagulation, inflammation, proliferation and remodeling [6]. For a patient with burns, microorganisms can penetrate into the tissues under the skin, developing chronic and systemic complications [7,8]. Excessive reactive oxygen species also have a negative role on the healing process [9]. Phytochemicals (alkaloids, terpenes, polyphenols, saponins and essential oils) can optimize the healing process through their antimicrobial, anti-inflammatory and antioxidant activities [6]. Phytochemicals are increasingly sought after as alternatives to synthetic drugs. In addition to their targeted effects, synthetic drugs cause adverse side effects to the human body. Well-known classes of drugs may result in certain levels of nephrotoxicity and hepatotoxicity [10]. Nonsteroidal anti-inflammatory drugs are among the medications used to treat pain, fever and inflammation; however, these drugs can cause significant toxicity [11]. According to previous works, several species belonging to the *Asteraceae* family have been studied for their healing effect [12,13,14], but the species *B. cinerea* has not yet been evaluated.

In the Saharo-Sindian region, herbaceous plants constitute a large proportion of plants used in traditional medicine. *B. cinerea* is traditionally used for treatment of colic, diarrhea, cough, rheumatism, digestive disorders, urinary tract infections, lung infections, fever and headaches [15]. Extracts of this species are also used for their antimicrobial and insecticidal activities. Essential oil of *B. cinerea* can be used as a biopesticide during integrated pest management against the cowpea weevil, *Callosobruchus maculatus* [16]. Exposure to either of the two extracts of *B. cinerea*, ethyl ether or ethyl acetate, for 24 h was active against the mosquito vector of malaria, *Anopheles labranchiae*, with the LC_50s_ ranging from 28 to 325 ppm [17]. The dichloromethane extract obtained from the aerial parts of *B. cinerea* shows antibiotic activity against the enteric bacterium, *Enterococcus faecalis* [18]. Extracts of *B. cinera* in n-butanol, ethanol, ethyl acetate and petroleum ether at 70% show antimicrobial activity against the bacteria *Escherichia coli* (*E. coli*), *Pseudomonas aeruginosa* (*P. aeruginosa*), *Staphylococcus aureus* (*S. aureus*) and *Klebsiella pneumoniae* [19]. The aqueous extract of *B. cinerea* inhibited two species of fungi of the genus *Fusarium* that affect yields of wheat [20].

*B. cinerea* has antipyretic effects and reduces fever [21]. The administration of an extract obtained from the dry plant material of *B. cinerea* reduced fever in rats in groups treated with 0.2 and 0.4 g/kg of their body mass (bm). Extracts of *B. cinerea* can be used for treatment of pain, which is defined as a rather uncomfortable physical sensation caused by disease or injury [22].

The present study characterized phytochemically the extracts from the aerial parts of *B. cinerea*, collected from the city of Akka, by the determination of their total polyphenol content (TPC), total flavonoid content (TF) and condensed tannins (CT) content by the use of chromatography and by high performance liquid chromatography, coupled with a diode-array detector (HPLC-DAD). To evaluate their pharmacological potential, in vivo analgesic activity, anti-inflammatory activities and, for the first time, their wound healing potential were characterized. Furthermore, in vitro antioxidant and antibacterial capacities were tested by various methods.

## 2. Materiel and Methods

### 2.1. Preparation of BCAE and BCEE

**Figure 1:**
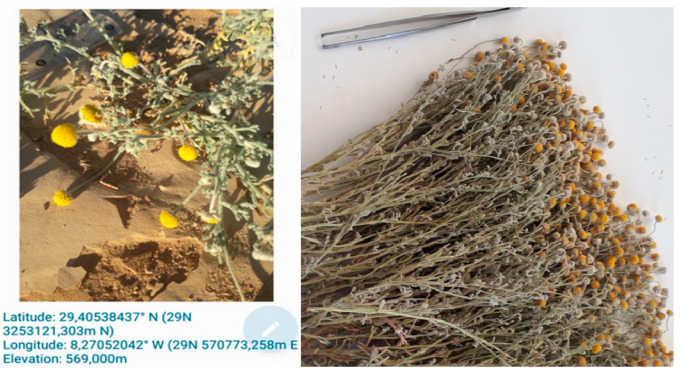
Aerial parts of *B. cinerea* in the flowering stage.

The plant studied (Figure 1) was collected during the second week of February 2021 in the city of Akka, Tata province in southeastern Morocco (latitude: 29.40538437° N (29 N 3253121,303 m N), longitude: 8.27052042° W (29 N 570773,258 m E), elevation: 569,000 m)). The sample was identified by the botanist Professor Bari Amina (Faculty of Sciences, Dhar El Mahraz, University of Sidi Mohamed Ben Abdellah, Fez), the voucher specimen was BC0019220211. In the preparation of the extracts (BCEE and BCAE), 100 g of the powder of the aerial parts (ground by a Waring^®^ blender, New Hartford, CT, USA) was macerated in 1 L of ethanol (Sigma-Aldrich, St. Louis, MO, USA) at 70% for 48 h, while the aqueous extract was prepared by infusion (100 g of the powder in 1 L of boiling water). The filtrates were dried at 37 °C until the total removal of the solvent and then stored at 4 °C [23].

### 2.2. Characterization of Phytochemicals

#### 2.2.1. Quantification of Total Polyphenol Content (TPC)

A volume of 500 μL of Folin reagent diluted 1/10 was added to 100 μL of the diluted sample. After 4 min, 400 μL of 75 mg sodium carbonate/mL was added to the reaction mixture. After 2 h incubation at laboratory temperature, the optical density was measured at 760 nm in a spectrophotometer. The concentrations of the total polyphenols were calculated by comparison to the calibration range equation established with Gallic acid (y = 5.3922x + 0.0122; R^2^ = 0.9933), expressed as milligram of Gallic acid equivalents per gram of extract (mg GAE/g extract) [24].

#### 2.2.2. Quantification of Total Flavonoids (Fl)

Flavonoids in extracts of *B. cinerea* were quantified by use of previously described methods [25] with slight modifications. A volume of 1.5 mL of the extract was added to 1.5 mL of AlCl3 (2%). The absorbance was measured after 30 min of incubation in the dark spectrophotometrically at 430 nm and compared to a blank and a standard curve (Y = 13.998X + 0.1133; R^2^ = 0.99). The total flavonoids were expressed as mg quercetin equivalents per g extract (mg QE/g extract).

#### 2.2.3. Quantification of Condensed Tannins (CT)

Concentrations of condensed tannins (CT) in extracts of *B. cinerea* were determined by spectrophotometry according to the previously described methods [26]. One hundred (100) µL of the extract was mixed with 500 µL of Folin–Ciocalteu and 1 mL of sodium carbonate (7.5%). After 30 min of incubation in the dark, the absorbance was measured at 760 nm. The concentrations of condensed tannins of the studied extracts were expressed as milligrams of tannic acid equivalence (TAE) per g of extract (mg TAE/g extract) according to an external standard curve (y = 5.615x − 0.0005; R^2^ = 0.9871).

#### 2.2.4. HPLC-DAD Analysis

##### Chemicals and Standard Preparations

The molecules used as the standards in this analysis were: caffeic acid, gallic acid, rutin, vanillin, quercetin, catechin, arbutin, rosmarinic acid, luteolin and furilic acid (Sigma-Aldrich, Roedermark, Germany). Methanol was the solvent used to prepare the standards at 100 ppm.

##### HPLC-DAD Conditions

In the reverse phase chromatographic analysis, a Shimadzu HPLC system with an analytical column (C18) SGE 250 × 4.6mm SS Exsil ODS 5 μm was used. A gradient mode separation was completed by using two solvents (X (water/acetic acid) (97.5: 2.5, *v*/*v*); Y (methanol/acetonitrile) (50: 50, *v*/*v*)), the injection volume was 20 µL and the flow rate was 1 mL/min. Wavelengths used for detection ranged from 200 to 800 nm. The elution gradient was: at 0 min (5% X, 95% Y); at 6.25 min (30% X, 70% Y); at 12.5 min (35% X, 65% Y); at 16.25 min (70% X, 30% Y); at 17.5 min (100% X, 0% Y); at 18.75 min (5% X, 95% Y) [27].

### 2.3. In Vitro Antioxidant Activity

#### 2.3.1. Trapping of Free Radicals by 2,2′-Diphenyl 1-Picrylhydrazyl Radical (DPPH)

The antioxidant potency of the extracts was measured as a hydrogen donor or free radicals, using stable 2,2’-diphenyl 1-picrylhydrazyl radical 4 mg DPPH/100 mL in 10 test tubes; 100 µL of the various concentrations of the aqueous extract, ethanolic extract and quercetin was added to 750 mL of the DPPH solute on [28]. After 20 min in the dark at room temperature, the absorbance was measured at 517 nm. Quercetin was used as the standard solution. The percentage of DPPH radical inhibition was calculated (Equation (1)).

(1) $$Inhibition\%=\left(Ab-Aa/Ab\right)\times 100$$

where Ab is the absorption of control (−) and Aa is the absorption of the extract.

#### 2.3.2. Ferric Reducing Antioxidant Power Assay (FRAP)

The ferric reducing power of the extracts was determined by the previously described methods [29]. Briefly, 200 µL of defined concentrations of the extracts was mixed with 500 µL of the phosphate buffer (0.2 M, pH 6.6) and 500 µL of potassium ferricyanide [K_3_Fe(CN)_6_] 1%. The resulting solution was incubated at 50 °C for 20 min, the mixture was acidified with 500 µL of 10% trichloroacetic acid (TCA), 0.5 mL of the supernatant was mixed with 500 µL of distilled water and 100 µL of Fe Cl3 (0.1%) and the absorbance was measured spectroscopically at 700 nm.

#### 2.3.3. Quantification of Total Antioxidant Capacity (TAC)

A total volume of 0.25 mL of a known concentration was added to 2.5 mL of the reagent solution (sulfuric acid (0.6 mol/L); sodium phosphate (28 mmol/L) and ammonium molybdate (4 mmol/L)). The mixtures were incubated in a water bath at 95 °C for one h and 30 min. After incubation, mixtures were cooled to laboratory temperature. An optical density measurement was made without a wavelength at 695 nm. The expression of the total antioxidant capacity of BCEE and BCAE was made by equivalents in mg of ascorbic acid per gram of extract (mg AAE/g extract) [30].

### 2.4. Antibacterial Activity

#### 2.4.1. Tested Strains

The antibacterial activities of the aqueous and ethanolic extracts of *B. cinerea* were evaluated against three bacterial strains, including the Gram-negative (*E. coli* (ATB:97) BGM and *pseudomonas aeruginosa*, as well as the Gram-positive *Bacillus subtills*, which were provided by the Bacteriology Laboratory of the Centre Hospitalier Universitaire Hassan II of Fez, Morocco.

#### 2.4.2. Disk Diffusion Method

The antibacterial efficacies and potencies of BCAE and BCEE were performed by adaptations of previously published methods [31]. Petri dishes containing a nutrient broth were inoculated with the three bacterial strains. Whatman paper discs (6 mm diameter) were placed on the surface of the inoculated media and impregnated with 0.02 mL of the extract diluted in DMSO at 100 mg extract/mL [32]. The inoculated Petri dishes were incubated at 37 °C, and the inhibition diameter measurement was performed after 24 h.

#### 2.4.3. Determination of the Minimum Inhibitory Concentration

The minimum inhibitory concentrations of the *B. cinerea* extracts against the three bacterial strains were revealed using the microdilution method according to the previously described methods [33]. After 24 h of incubation at 37 °C, the MIC endpoint was determined by the direct observation of the growth in the wells and by using the colorimetric method (TTC 0.2% (*w*/*v*)) [34,35].

### 2.5. Pharmacological Activities

#### 2.5.1. Animal Handling and Housing

In order to characterize anti-inflammatory potency, analgesic and wound healing activity, male mice (0.032 to 0.045 kg) and male rats (0.12 to 0.14 kg) were obtained from the Department of Biology, Faculty of Sciences Dhar El-Mahraz, Sidi Mohamed Ben Abdellah University, Fez, Morocco. The animal housing conditions were: day/night photoperiod (~12/12 h); temperature (28–32 °C); humidity (50–55%). The animals were given free access to food and water, adapted and handled with ethics committee approval (July 2020/LBEAS-08 and 25 February 2021).

#### 2.5.2. Wound Healing Test

##### Formulation of Ointment

**Figure 2:**
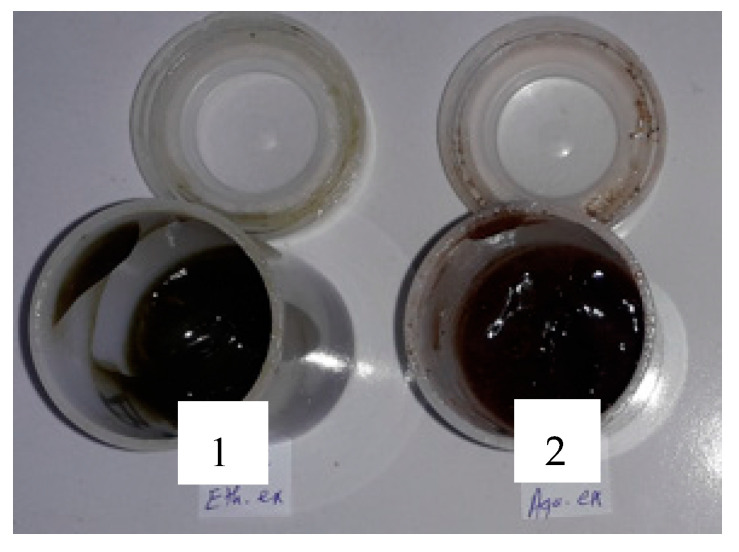
Ointment from BCEE (1) and BCAE (2).

Ointments were prepared from aqueous and ethanolic dry extracts of the aerial parts of *B. cinerea* at 10% in Vaseline^®^ by gentle trituration until the mixture was homogenized. The resulting ointments (Figure 2) were packed in hermetically sealed jars and stored until use.

##### Burn Wound Induction

Wound induction was performed by burning the dorsal area, using the methods described in previous work [36] with slight modifications. Twenty Wistar rats divided into four groups were used: a negative control group (Vaseline^®^ treatment), a second group treated with a healing ointment (Madecassol^®^ at 1%), a third group and a fourth group treated with the BCAE and BCEE ointments, respectively. An electric tensioner was used to shave the dorsal area of the rats, then an intraperitoneal anesthesia (pentobarbital at 50 mg/kg) was conducted before the induction of the burn (placement of a 1.7 cm diameter aluminum rod heated in boiling water on the burn induction area for 10 s). The treatment over the three weeks was performed by the daily covering of the burned dorsal surfaces with the prepared ointments. Photographs were taken of the wound surfaces using a digital camera with the presence of a graduated ruler. The images obtained were analyzed using ImageJ software in order to measure contraction rate of the wound surfaces (Equation (2)).

(2) $$WC\;\left(\%\right)=\left[\left(WS0-WSSD\right)/WS0\right]\times 100$$

where WC is the wound contraction, WS0 is the size of the wound on the first day and WSSD is the wound size on each specific day.

#### 2.5.3. Carrageenan-Induced Inflammation of the Right Rat Paw

Evaluation of anti-inflammatory power of BCEE and BCAE (extracts obtained from the aerial parts of *B. cinerea*) was carried out according to the protocol described [37]. Twenty Wistar rats divided into four groups were used in this test: one group received BCEE 500 mg/kg orally, a second group received BCAE 500 mg/kg, a negative control group received 0.9% NaCl and a positive control group received Indomethacin^®^ (10 mg/kg). A measurement of the circumference of the right paw, of each rat was carried out before the injection of the suspension of carrageenan (1%), then measurements of the circumferences of the paws were carried out after the third, fourth, fifth and sixth hour of the injection. An equation was used to calculate the percentage of inflammation inhibition (Equation (3)).

(3) $$PI\%=\left[\left(Ct-C0\right)\;Control-\left(Ct-C0\right)\;treated/\left(Ct-C0\right)\;Control\right]\times 100$$

where C0 is the mean circumference of the paw before injection and Ct is the mean circumference of the paw after a carrageenan injection at a given time.

#### 2.5.4. Analgesic Activity

Four batches, each containing five mice of different weights (32 to 45 g) were used in this study. BCAE, BCEE and the reference drug (Tramadol^®^) were administered orally to the mice at a dose of 500 mg/kg, bm. The control mice received only physiological water (NaCl 0.9%). After 1 h 30 min, a 0.5% acetic acid solution was injected intraperitoneally (10 mL/kg). Subsequently, 5 min after the acetic acid injection, nociception was assessed for 30 min by counting the number of abdominal contortions [38] (Equation (4)).

(4) $$PI\%=\left(\left(Mn-Mt\right)/Mn\right)\times 100$$

where Mn is the mean number of abdominal contractions (negative control group) and Mt is the average number of abdominal contractions (group treated with the extracts or standard compound).

#### 2.5.5. Statistical Analyses

The results were analyzed with the one-way ANOVA test (GraphPad Prism software version 8), and *p* < 0.05 showed statistical significance. The results are expressed as mean ± SD.

## 3. Results and Discussion

### 3.1. TPC, Fl, CT and HPLC-DAD Analysis

**Figure 3:**
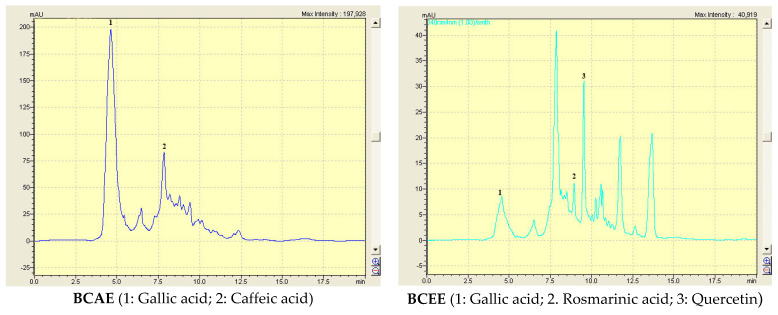
Chromatograms of BCAE and BCEE obtained by HPLC-DAD analysis.

The yield obtained by maceration using ethanol (27.34%) was greater than that obtained by diffusion in water (17.50%). The concentrations of TPC, Fl and CT in the extracts of *B. cinerea* extracts were determined (Table 1). The concentrations of phenolic compounds were greater in the ethanolic extract than in the aqueous extract (21.06 and 13.09 mg of GAE/g extract, respectively, for BCEE and BCAE). These extracts are characterized by the presence of large amounts of condensed tannins expressed by mg tannic acid equivalents/g extract; this amount was 24.05 mg in the ethanolic extract. The analysis of BCAE and BCEE by HPLC-DAD allowed the detection of four phenolic compounds (gallic acid and caffeic acid in BCAE; gallic acid, rosmarinic acid and quercetin in BCEE) (Table 2 and Figure 3). Even if there are molecules that are not revealed, the above mentioned compounds were among the main molecules of the *B. cinerea* extracts.

The compositions of TPC, Fl and CT in the extracts of *B. cinerea* were variable. According to Chlif et al. (2022) [39], the aqueous extract obtained from the aerial parts of *B. cinerea* contains 23.03 mg of GAE/g extract, 11.69 mg of QE/g extract and 6.41 mg of TAE/g extract. Previous studies have found methanolic extracts in TPC, expressed as mg GAE/g of dry matter (dm), Fl, expressed as mg QE/g of dm and condensed tannins, expressed as mg cyanidin equivalent/g of dm, which were 2.95, 44.58 and 1.44, respectively [40]. In another study, the contents were 22.22 mg GAE/g of dm, TPC 3.93/g of dm and 8.61 catechin equivalents/g of dm for Fl and proanthocyanides, respectively [41]. Among the main phenolic compounds revealed by HPLC-ESI MS in a methanolic extract of the aerial parts of *B. cinerea* are luteolin-4’-*O*-glucoside, 3,5-dicaffeoylquinic acid, 4,5-dicaffeoylquinic acid, 3,4-dicaffeoylquinic acid, cryptochlorogenic acid and chlorogenic acid [42]. The hydroalcoholic extract of the aerial parts of *B. cinerea* contains other phenolic compounds (flavonoid compounds), including chrysospenol-D, chrysosplenetin, oxyayanin-B, axillarin, 3-methylquercetin, pedaletin, isokaempferid, apigenin, luteolin, 6-hydroxyluteolin and others [43]. The remarkable differences in polyphenols between the studied extracts and other extracts reported in the bibliography are strongly explained by the intervention of several factors, among which are the geographical situation and the climate of the harvesting station. The content of phytochemicals, including polyphenols, varies under the action of water deficit (drought stress); this factor leads to differences in the accumulation of polyphenols in the different organs of several plant species [44,45,46].

### 3.2. Antioxidant Activity (TAC, DPPH and FRAP Tests)

Extracts of *B. cinerea* (BCEE and BCAE) showed a remarkable antioxidant capacity, (Table 3). The BCEE extract showed a higher total antioxidant capacity than BCAE, 5.312 ± 0.217 mg of AAE/g extract and 2.832 ± 0.148 mg of AAE/g extract, respectively. BCAE was very active against the DPPH radical (IC_50_ = 4.9 × 10^−2^ mg/mL), but this antiradical power was less than that obtained using Quercitin (7.9 × 10^−4^ mg/mL). BCEE was the least active against DPPH. Moreover, results of the FRAP assay revealed that BCEE has a moderate ability to reduce iron, while BCAE appeared to be inactive. The richness of BCEE in polyphenolic compounds with high antioxidant power could explain the obtained results.

Comparing the results obtained in other studies, the methanolic extract of *B. cinerea* has a total antioxidant capacity of 17.190 ± 1.273 mg ascorbic acid equivalents/g of dm, while this extract has a DPPH free radical scavenging effect (EC_50_ = 462.19 mg antioxidant/g DPPH), hydroxyl radical scavenging (EC_50_= 0.66 ± 0.12 mg/mL) and iron reducing capacity (IC_50_ = 1.174 ± 0.05 mg/mL) [41]. Pure polyphenolic compounds isolated from the aerial parts of *B. cinerea* exert antioxidant activity assessed by the use of the DPPH assay; IC_50_s are expressed as (µM): luteolin-4’-*O*-glucoside (30.25), 4,5-dicaffeoylquinic acid (31.49), 3,5-dicaffeoylquinic acid (23.84), 3,4-dicaffeoylquinic acid (30.25), neochlorogenic acid (11.0) and chlorogenic acid (10.5) [47]. In addition, the essential oil of *Cotula cinerea* (a synonym of *B. cinerea*) showed antioxidant activity measured by different assays DPPH, PRAP, B-carotene and ABTS with an IC_50_ (mg/mL) of 1.1 ± 0.15, 2.7 ± 0.09, 0.63 ± 0.16 and 0.91 ± 0.52, respectively [48].

### 3.3. Antibacterial Activity of BCAE and BCEE

**Figure 4:**
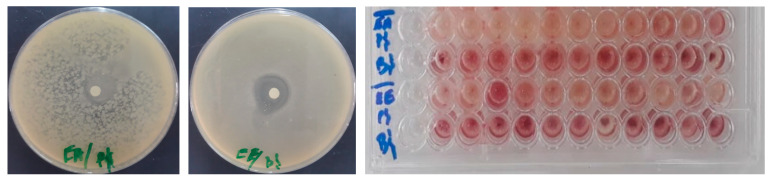
Photos showing the antibacterial effect of extracts of *B. cinerea*.

Extracts obtained from the aerial parts of *B. cinerea* exhibited antibacterial activity against both gram-negative and gram-positive bacteria (Table 4 and Figure 4). BCEE was more active against *B. subtills* with a zone of inhibition of 23.16 mm, while BCAE was active against the gram-negative bacteria *P. aeruginosa* (the diameter of the zone of inhibition was 18.66 mm); the MIC values for both the prevalent bacteria were 10 mg/mL. However, *E. coli* was resistant to the extracts used in this study. The MIC values obtained by using streptomycin against the strains used were 25 ± 1.63 and 2.81 ± 0.095 mg/mL, respectively, for *E. coli* and *B. subtilis*, while *P. aeruginosa* was resistant to this antibiotic [16].

Compared with the results of previous studies, aqueous extracts obtained from aerial parts, leaves and flowers of *B. cinerea* showed potent activity against gram-positive bacteria (*S. aureus* and *S. faecalis*) with MICs ranging from 0.78 to 12.5 mg/mL [39]. Alternatively, the gram-negative bacteria, *E. coli* and *K. pneumoniae*, were moderately sensitive to the extracts tested, with MICs ranging from 3.13 to 50 mg/mL, with the exception of *P. aeruginosa*, which showed resistance to extracts of the various parts of *B. cinerea. Enterococcus faecalis,* a gram-positive bacterium, was inhibited by a dichloromethane extract of the aerial parts of *B. cinerea* [18]. In another study, the inhibition zones obtained against *S. aureus* by using *B. cinerea* extracts were 12 ± 5.20 mm and 11.67 ± 3.79 mm for the n-butanol and ethyl acetate extracts, respectively, whereas the hydroalcoholic extract showed weak antimicrobial activity against the tested strains [19].

Part of the antimicrobial activity exhibited by the extracts obtained from the aerial parts of *B. cinerea* is the result of the antibacterial effect of some phenolic compounds revealed in the studied extracts. Gallic acid may be involved in disrupting the integrity of the membranes of bacterial strains, modifying hydrophobicity, permeability and surface charge [49]. Rosmarinic acid exhibits antimicrobial activity against gram-positive strains. Thus, the lowest blocking concentration against *S. aureus* was 0.8 mg/mL, and the methicillin-resistant *S. aureus* strain was blocked by a concentration of 10 mg/mL [50]. Caffeic acid and other phenylpropanoids have inhibited the growth of certain bacterial strains, including *S. aureus*, *E. coli*, *Listeria monocytogenes* and *Bacillus cereus* [51]. Quercitin can inhibit bacterial growth; its ability to inhibit D-Ala-D-Ala ligation in bacterial cells gave it a bacteriostatic activity [52]. According to Ferreira et al. (2013) [53], in the majority of cases, hydroxybenzoic acids exerted weak antibacterial effects compared to hydroxycinnamic acids.

### 3.4. Pharmacological Activities

#### 3.4.1. Wound Healing Activity of *B. cinerea* Extracts

**Figure 5:**
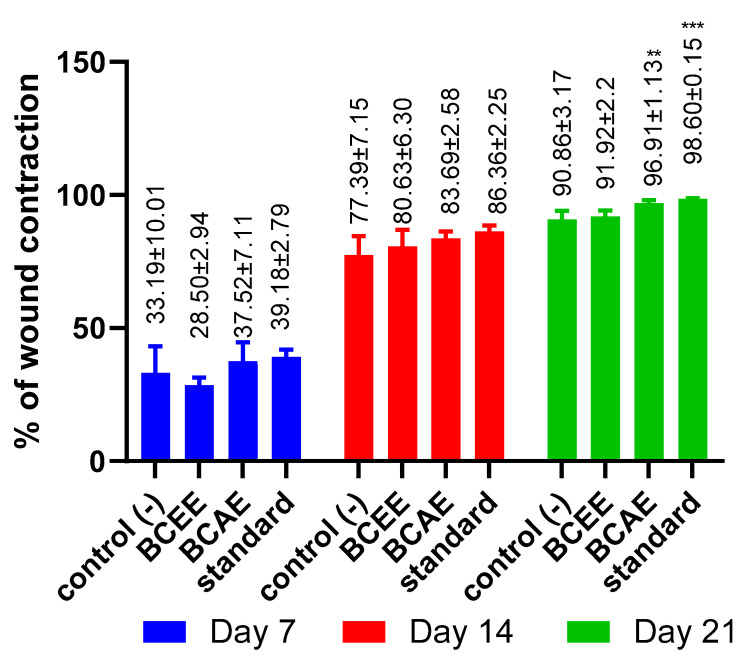
% of wound contraction using *B. cinerea* extracts and standard treatment (Indomethacin). (Dunnett’s multiple comparisons test relative to the control: ** *p* < 0.01; *** *p* < 0.001).

**Figure 6:**
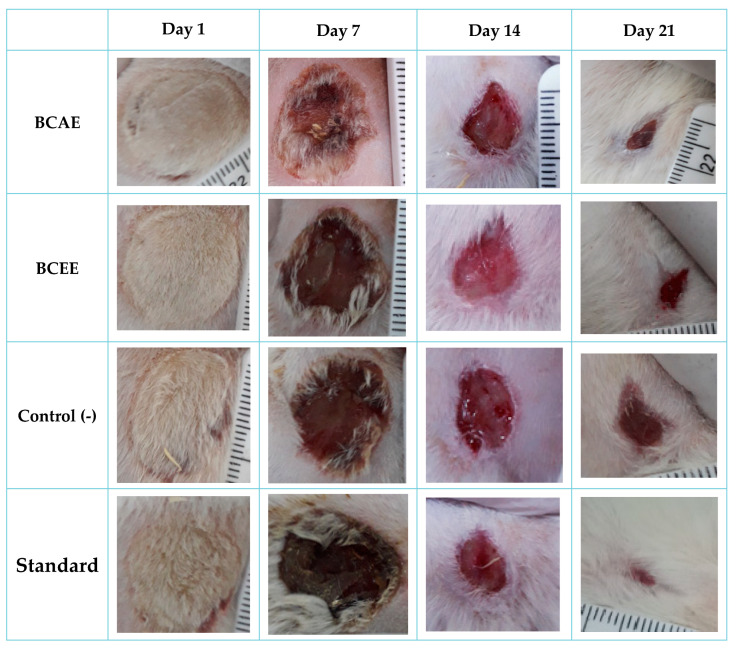
Images of the healing process in treated rats.

Wound healing ability of *B. cinerea* extracts was examined in the Wistar rat burn model. The rate of wound healing in the different groups was different (Figure 5), and the images showing the healing process are shown in Figure 6. The BCAE-based pomade treatment showed a higher rate of healing compared to that obtained by BCEE or by the negative control (Vaseline treatment). At the end of the third week of treatment, the BCAE-based pomade resulted in a very remarkable healing rate (96.91%) close to that of the positive control (98.60%) (*p* value < 0.05), whereas this healing rate was only 90.86% in the negative control group.

To our knowledge, this is the first study to report on the healing activity of extracts of *B. cinerea,* which have not been reported among the 12 species belonging to the Asteraceae family—species that are part of medicinal plants widely used in the treatment of wounds in the Mediterranean region [54]. An evaluation of the healing activity of a plant species belonging to the asteraceae family and using the same burn model with an extract of *Ditrichia viscosa* shows a remarkable wound contraction (99.28%) after 21 days of treatment [27]. The healing efficacies of some species of asteraceae can be attributed to their antibacterial effects, which provide protection of the microenvironment and damaged wound tissue against pathogenic bacterial strains [55].

#### 3.4.2. Anti-Inflammatory Activity

**Figure 7:**
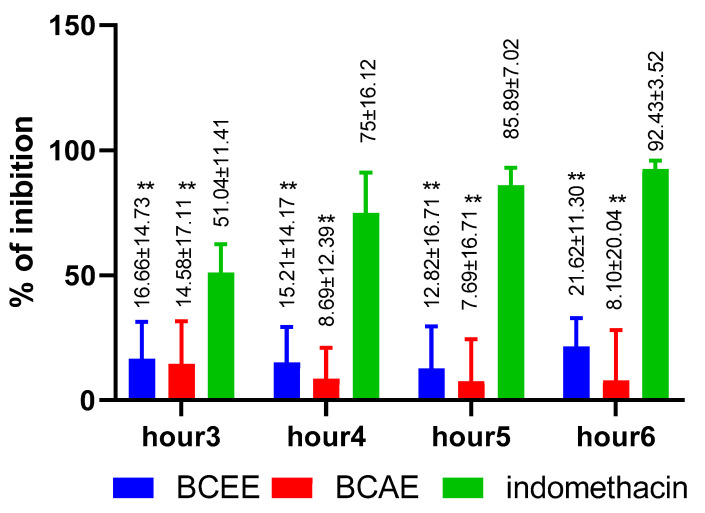
Anti-inflammatory activity of *B. cinerea* extracts and the standard compound (Dunnett’s multiple comparisons test relative to the standard: ** *p* < 0.01).

The anti-inflammatory efficacy and potency of the studied extracts was less than that of the positive control of Indomethacin (Figure 7). Despite its richness in different phenolic types, BCAE has minimal anti-inflammatory efficacy with the greatest % inhibition of 8.1% observed during the sixth hour. BCEE exhibited greater anti-inflammatory efficacy than BCAE. The percentage of inhibition obtained using BCEE varies between 12.82% and 21.62% during the 3 h of measurement (*p* value < 0.01).

There are few studies on the anti-inflammatory efficacy or potency of extracts of *B. cinerea*. At a dose of 400 mg/kg bm, aqueous extracts of fresh and dry aerial parts of *B. cinerea* inhibited inflammation by 47.73% and 50.01%, respectively, 5 h after a carrageenan injection, compared to the standard drug, Indomethacin (55.26%) [21]. In another study, the measurement of the anti-inflammatory effect using murine macrophage-like RAW 264.7 cells and quantified by nitric oxide (NO) production shows that the infusion and hydroethanolic extracts revealed a weaker inhibition of NO production, with EC_50_ values of 122 ± 6 and 105 ± 9 µg/mL, respectively [56]. Iluteolin, apigenin, kaempferol and caffeoylquinic acid derivatives are known to have a potent anti-inflammatory effect [57,58].

#### 3.4.3. Peripheral Analgesic Activity

**Figure 8:**
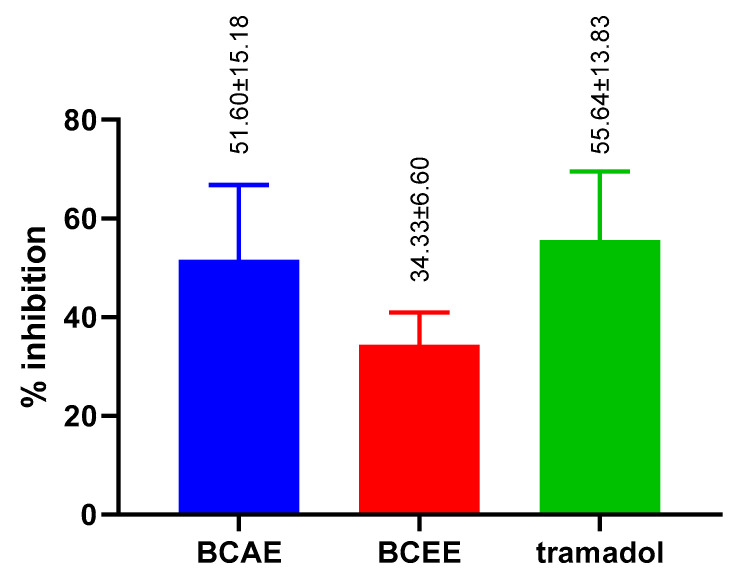
Analgesic efficacy of extracts of *B. cinerea* and the standard compound, Tramadol (Bartlett’s statistic (corrected): *p* value = 3031; are SDs significantly different: no).

BCAE exerts remarkable analgesic activity with a percentage of inhibition of 51.60 **±** 15.18%, while BCEE exerts weak activity with a percent of inhibition of 34.33 ± 6.60%. BCEE also shows low analgesic activity compared to Tramadol (55.64 ± 13.83%) with a significant difference (Figure 8). BCAE exhibited analgesic activity, similar to that of Tramadol with no significant difference.

The results of a recent study found that a dose of 400 mg/kg, bm of aqueous extracts of fresh and dry aerial parts of *B. cinerea* were effective at inhibiting spasms caused by the injection of acetic acid (0.6%) by 50.71% and 45.51%, respectively [21]. Alternatively, it has been reported that the use of n-butanol, ethyl acetate and ethyl ether extracts induced a percentage of inhibition of constriction of 40.21%, 50% and 62.49%, respectively [59]. The analgesic efficacies of extracts of fresh and dry aerial parts of *B. cinerea* could be related to an alteration of the biosynthesis of the prostaglandins by the inhibition of the cyclooxygenase [21].

## 4. Conclusions

The chemical composition of extracts of *B. cinerea* is consistent with its use as a traditional pharmacological treatment. The application of BCAE and BCEE on rats and mice demonstrated the ability of the aqueous extract to minimize abdominal contortions and to increase the rate of re-epithelialization of burn-induced wounds. The healing power revealed by this study, through the use of the aqueous extract of *B. cinerea*, promotes the application of this extract in the development of treatments that combine the use of modern products and practices with herbal healing agents. Moreover, the results of the antioxidant activities showed that the ethanolic extract is recommended as an antioxidant agent due to its high content of polyphenols, flavonoids and tannins. Thus, this work provides useful results for further pharmacological studies and the design of new drugs based on the species of *B. cinerea.*

## Figures and Tables

**Table 1:** Concentrations of total phenolic content (TPC), total flavonoids (Fl) and condensed tannins (CT) in extracts of *B. cinerea*.

	TPC: mg GAE/g of Extract	Fl: mg QE/g of Extract	CT: mg TAE/g of Extract
BCAE	13.09 ± 0.13	9.86 ± 0.41	17.07 ± 0.36
BCEE	21.06 ± 0.04	10.43 ± 0.13	24.05 ± 0.24

**Table 2:** Compounds identified in *B. cinerea* extracts and chromatographic retention times (RT).

Phenolic Compounds	RT (min)	DO (nm)
Gallic acid	4.69	280
Caffeic acid	7.777	300
Rosmarinic acid	8.300	320
Quercetin	9.403	370

**Table 3:** Antioxidant activity of extracts of *B. cinerea*.

TAC (mg AAE/g Extract)		FRAP (EC_50_ mg/mL)			DPPH (IC_50_ mg/mL)		
BCEE	BCAE	BCEE	BCAE	Quercitin	BCEE	BCAE	Quercitin
5.312 ± 0.217	2.832 ± 0.148	0.248668	-	0.007295	0.144	0.049	0.000799

**Table 4:** Antibacterial activity of *B. cinerea* extracts.

	Zone of Inhibition (mm)			CMI mg/mL		
	*P. aeruginosa*	*E. coli*	*B.* *subtils*	*P. aeruginosa*	*E. coli*	*B.* *subtils*
BCEE	-	-	23.16 ± 0.76	-	-	10 ± 0.0
BCAE	18.66 ± 1.6	-	-	10 ± 0.0	-	-

## Data Availability

All data pertinent to the work are included in the paper.
